# Elevated serum IL-10 is associated with severity of neonatal encephalopathy and adverse early childhood outcomes

**DOI:** 10.1038/s41390-021-01438-1

**Published:** 2021-03-05

**Authors:** Raymand Pang, Brian M. Mujuni, Kathryn A. Martinello, Emily L. Webb, Angela Nalwoga, Julius Ssekyewa, Margaret Musoke, Jennifer J. Kurinczuk, Margaret Sewegaba, Frances M. Cowan, Stephen Cose, Margaret Nakakeeto, Alison M. Elliott, Neil J. Sebire, Nigel Klein, Nicola J. Robertson, Cally J. Tann

**Affiliations:** 1 Institute for Women’s Health, University College London, London, UK; 2 Medical Research Council/Uganda Virus Research Institute and London School of Hygiene and Tropical Medicine Uganda Research Unit, Entebbe, Uganda; 3 MRC International Statistics and Epidemiology Group, London School of Hygiene and Tropical Medicine, London, UK; 4 National Perinatal Epidemiology Unit, University of Oxford, Oxford, UK; 5 Department of Pediatrics, Imperial College London, London, UK; 6 Department of Clinical Research, London School of Hygiene & Tropical Medicine, London, UK; 7 UCL Institute of Child Health and GOSH BRC, UCL, London, UK; 8 Centre for Clinical Brain Sciences, University of Edinburgh, Edinburgh, UK; 9 Department of Infectious Disease Epidemiology, London School of Hygiene and Tropical Medicine, London, UK

## Abstract

**Background:**

Neonatal encephalopathy (NE) contributes substantially to child mortality and disability globally. We compared cytokine profiles in term Ugandan neonates with and without NE, with and without perinatal infection or inflammation and identified biomarkers predicting neonatal and early childhood outcomes.

**Methods:**

In this exploratory biomarker study, serum IL-1α, IL-6, IL-8, IL-10, TNFα, and VEGF (<12 h) were compared between NE and non-NE infants with and without perinatal infection/inflammation. Neonatal (severity of NE, mortality) and early childhood (death or neurodevelopmental impairment to 2.5 years) outcomes were assessed. Predictors of outcomes were explored with multivariable linear and logistic regression and receiver-operating characteristic analyses.

**Results:**

Cytokine assays on 159 NE and 157 non-NE infants were performed; data on early childhood outcomes were available for 150 and 129, respectively. NE infants had higher IL-10 (*p* < 0.001), higher IL-6 (*p* < 0.017), and lower VEGF (*p* < 0.001) levels. Moderate and severe NE was associated with higher IL-10 levels compared to non-NE infants (*p* < 0.001). Elevated IL-1α was associated with perinatal infection/inflammation (*p* = 0.013). Among NE infants, IL-10 predicted neonatal mortality (*p* = 0.01) and adverse early childhood outcome (adjusted OR 2.28, 95% CI 1.35–3.86, *p* = 0.002).

**Conclusions:**

Our findings support a potential role for IL-10 as a biomarker for adverse outcomes after neonatal encephalopathy.

**Impact:**

- Neonatal encephalopathy is a common cause of child death and disability globally. Inflammatory cytokines are potential biomarkers of encephalopathy severity and outcome.
- In this Ugandan health facility-based cohort, neonatal encephalopathy was associated with elevated serum IL-10 and IL-6, and reduced VEGF at birth.
- Elevated serum IL-10 within 12 h after birth predicted severity of neonatal encephalopathy, neonatal mortality, and adverse early childhood developmental outcomes, independent of perinatal infection or inflammation, and provides evidence to the contribution of the inflammatory processes.
- Our findings support a role for IL-10 as a biomarker for adverse outcomes after neonatal encephalopathy in a sub-Saharan African cohort.

## Introduction

Intrapartum-related deaths are the third leading cause of global neonatal mortality.^1^ Complications around the time of birth, leading to neonatal encephalopathy (NE), have been estimated to affect 1.15 million babies globally each year and contribute to many more with neurodevelopmental impairment (NDI),^2^ including cerebral palsy (CP), global developmental delay, vision and hearing impairments, and seizure disorders.^3^ NE is defined as a syndrome of impaired neurological function in the first few hours and days after birth. Symptoms include reduced consciousness, abnormal tone and reflexes, seizures, and difficulty maintaining respiration.^4^ The large majority (96%) of NE cases occur in low- and middle-income countries (LMICs), with the greatest burden in sub-Saharan Africa.^2^ In resource-limited regions such as sub-Saharan Africa, being able to identify infants with NE who are at greatest risk of death and disability could support targeted therapeutic early intervention.

Cytokines and chemokines are potential biomarkers of NE severity and outcome. Inflammation plays a key role in intrapartum-related NE.^5,6^ An inflammatory cascade in response to hypoxic–ischemic (HI) injury is well recognized and contributes to both brain injury and repair.^5,6^ In addition, preclinical and observational data suggest that perinatal infection sensitizes the newborn brain to HI, increasing the risk of NE and resulting mortality and long-term neurological injury.^7–9^ Neuroinflammation is characterized by microglial activation, cytokine and chemokine expression, with subsequent infiltration of immune cells and activation of cell death pathways.^5^ Cytokines have the potential to indicate the severity of neuroinflammatory response following NE and to assist in the detection of contributing co-infection.

Biomarkers to aid in the accurate prediction, diagnosis and prognostication for NE are actively sought across high- and low-resource settings. Validated outcome biomarkers available to clinicians in sophisticated neonatal intensive care settings, such as amplitude integrated electroencephalography^10^, magnetic resonance imaging (MRI) and spectroscopy,^11^ are not widely available in LMICs. Practical, cost-effective alternatives are required. Cytokine profiles in relation to NE have been studied over the past two decades, with cytokines in serum and cerebrospinal fluid variably reported to be predictive of neurodevelopmental outcome after NE.^12–15^ However, studies to date have been limited by small numbers, exclusion of infants with evidence of co-infection, and a lack of long-term outcome data. In addition, studies have been largely limited to high-resource settings.

We aimed to (1) determine the cytokine profile in term infants with NE compared to term infants without NE, (2) compare cytokine profile between NE infants with and without perinatal infection and inflammation, and (3) identify cytokine biomarkers for neonatal and early childhood outcomes. We hypothesized that cytokine levels differ with severity of brain injury and perinatal infection or inflammation and can therefore play a role in predicting neonatal and early childhood outcomes.

## Methods

The study was approved by the Uganda Virus Research Institute Research Ethics Committee, Mulago Hospital Ethics Committee, London School of Hygiene & Tropical Medicine Research Ethics Committee, University College London, and the Uganda National Council for Science and Technology. The reporting of this study complies with the STROBE (Strengthening The Reporting of OBservational Studies in Epidemiology) guidelines.

### Setting

Uganda is a low-income country with a neonatal mortality rate of 27 per 1000 live births.^16^ At the time of the study, high-risk pregnancies were delivered at Mulago National Referral Hospital (MNRH) in Kampala (study recruitment site). In labor, fetal monitoring was by intermittent auscultation using a Pinard stethoscope, assisted deliveries (ventouse/forceps) were not routinely offered, and one-fifth of deliveries were by cesarean section (few are electively planned). Midwife-led neonatal resuscitation included oxygen and bag-mask ventilation. Routine care in the 70-bed Special Care Baby Unit included continuous positive airway pressure, intravenous fluids including glucose, antibiotics and anti-seizure medication, but not mechanical ventilation, therapeutic hypothermia, cerebral function monitoring, or brain imaging.

### Study design and participants

This was an exploratory biomarker study nested within the ABAaNA (“Abaana,” meaning “children” in the local language Luganda) study,^17^ a prospective unmatched case–control study investigating perinatal risk factors for NE among neonates born at MNRH between September 2011 and October 2012, followed by a cohort comparison study of recruited infants followed up to 2 years of age. The research methodology and primary results have been described previously.^17–20^ In summary, term infants (≥37 weeks gestation) with NE (Thompson score^21^ ≥6 within 12 h of birth) and an unmatched comparison group of term infants without NE were included. Control infants were contemporaneously recruited (at a case:control ratio of 1:2), systematically sampled from the labor ward admission book, and were eligible if their Thompson score was <3 (as assessed by trained MNRH study physicians). Exclusion criteria for both NE and non-NE infants included living >20 km from MNRH, being out-born, neonatal antibiotic administration prior to recruitment (rare), and no written informed parental consent.

### Serum cytokine specimen collection and processing

At the time of recruitment, 0.5 ml of serum for cytokine analysis was obtained from all infants and stored at −80 °C. Cytokine samples were processed for all infants with NE. For the comparison group, a subset of serum cytokine samples was selected at random using a table generated in Stata v11.2® (with one comparison group sample selected for every infant with NE) and processed. Samples were thawed and processed using multiplex cytokine immunoassay (Luminex) between 13 and 17 May 2014, during the neurodevelopmental follow-up phase of the study. Samples with <0.5 ml volume and any incorrectly labeled were excluded. Serum concentrations of interleukin (IL)-1 alpha (IL-1α), IL-6, IL-8, IL-10, tumor necrosis factor alpha (TNFα), and vascular endothelial growth factor (VEGF) were measured using Human premixed multi-analyte kits (R&D Systems, Minneapolis, USA) on the Bio Plex200 platform (Bio Plex™, Bio Rad Laboratories). Cytokine concentrations were obtained from the Bio Plex manager software. Cytokine concentrations below the detection limit were assigned zero and values above the detection limit were assigned the value of the highest concentration standard.

### Perinatal infection and inflammation

At recruitment, blood samples were obtained for blood culture, species-specific bacterial detection using quantitative polymerase chain reaction (qPCR), and c-reactive protein (CRP) measurement from all infants with NE and from control infants with a clinical suspicion of sepsis as previously published.^19^ Isolated colonies from blood culture were manually identified. Multiplex real-time qPCR assays for pathogenic bacteria among newborns (group B Streptococcus, Pneumococcus, *Staphylococcus aureus*, group A streptococcus, Enterobacteriaceae sp.) were performed.^13,15^ Neonatal bacteremia was defined as either positive isolated colonies on blood culture or positive detection from qPCR assay. Maternal and neonatal CRP were measured as a marker of inflammation and are presented according to centiles among control mothers and infants, respectively, consistent with the reporting of the main study findings.^13^

The examination of placental histology was not routine at MNRH. In the ABAaNA study, placental histology was reported in around a quarter of NE (60/210) and non-NE (102/409) infants.^13^ Whole placentas were collected and fixed in 10% formalin and specimens were processed according to standard protocols.^13^ Histology was reported by an experienced perinatal pathologist based at the Camelia Botnar Laboratories, Great Ormond Street Hospital, UK, blinded to all clinical details. Histological chorioamnionitis and funisitis were diagnosed according to standard criteria.^22^

### Neonatal outcomes

Severity of encephalopathy was graded (mild, moderate, or severe) daily between days 1 and 5 according to the modified Sarnat classification^23^ and the grading used was the most severe recorded. The Sarnat score was determined by the principal investigator based on the clinical variables collected. Neonatal death was defined as death in the first 28 days after birth.

### Early childhood outcomes

The procedure used in the current study for neurodevelopmental follow-up has been previously reported.^18^ In summary, children were assessed at 12–15 and 27–30 months of age, either in outpatient clinic or at home (<5%). Transport costs were remunerated. Further informed written consent was taken at these visits. In case of death, parental report of causation and date of death was obtained.

Neurodevelopmental assessment was performed by trained and certified assessors, blinded to the presence of NE, clinical history, and investigations. Griffith’s Mental Developmental Scales-II (GMDS) was used to calculate a global Development Quotient (DQ) from the six subscales (A–F). The Hammersmith Infant Neurological Examination (HINE) (https://hammersmith-neuro-exam.com) provided a standardized score, which has been validated as a predictor of motor outcome at 12–18 months but is frequently extended into use in the second year.^24,25^ CP was diagnosed according to the Surveillance of Cerebral Palsy in Europe classification^26^ as spastic bilateral, spastic unilateral, dyskinetic, dystonic, choreo-athetotic, ataxic, or non-classifiable. Videos for children with a suboptimal HINE score (<73) or any asymmetries were reviewed by a minimum of two blinded investigators with expertise in NDI and consensus was reached in all cases. NDI was defined as a global DQ < 70 on GMDS and/or HINE score <67 and/or diagnosis of CP.

### Statistical analysis

Statistical analysis was carried out using SPSSv24 (IBM) and Prism v8 (GraphPad, USA). Data were assessed for normality and, where necessary, log_10_ transformed to normalize the distribution. Where relevant, data were back transformed and presented as geometric mean ratios.

Characteristics of participants with and without cytokine data were compared using chi-squared and *t* tests. Crude comparisons of serum cytokine concentrations between non-NE infants and NE infants were initially done using Mann–Whitney *U* tests due to the non-normal distribution of some cytokines. A multivariable linear regression model was fitted for each log_10_ transformed cytokine, adjusting for time of sampling and infant sex to identify significant differences in the cytokine profiles between the groups after adjusting for these potential confounders. The multivariable linear regression model was also used to identify significant differences between NE infants with and without perinatal infection or inflammation, adjusted for infant sex and time of sampling. Neonatal CRP >97th centile, bacteremia, maternal CRP >90th centile and histological funisitis, previously identified as risk factors for NE in our parent study,^17^ were utilized in this study to define perinatal infection/inflammation.

For neonatal outcomes, cytokine levels for NE infants by the highest modified Sarnat score in the first 5 days were compared with those of non-NE infants using Kruskal–Wallis with post hoc Dunn–Bonferroni tests. The crude levels were also compared between NE infants who survived and those who died and a multivariable logistic regression analysis was carried out among infants with NE to assess the ability of cytokine levels to predict neonatal fatality, adjusting for time of sampling and infant sex.

For early childhood outcomes, adverse outcome was defined as death or NDI. Where possible, 2-year neurodevelopmental outcomes were used; however, where absent, 1-year outcome data were included to minimize bias. A random sample of non-NE infants with both 1- and 2-year outcome data were compared to ensure consistency between the two follow-up time points. Crude serum cytokine concentrations were compared between non-NE infants, NE infants with favorable outcomes, and NE infants with adverse outcomes using Kruskal–Wallis with post hoc Dunn–Bonferroni tests. A multivariable logistic regression model was fitted to identify the ability of cytokines to predict adverse early childhood outcomes among children with NE, adjusting for infant sex, time of sampling, and neonatal bacteremia. Receiver-operator characteristic curves were constructed to determine the predictive value of cytokines.

### Sample size

The sample size for this substudy was determined by the availability of serum samples from the original ABAaNA study. We carried out a post hoc power calculation based on the calculated standard deviations for each of the cytokine concentration values on the log_10_ scale in our dataset, which ranged from 1.02 to 2.88. Therefore, for the first objective of determining the cytokine profile in infants with NE compared with that of the comparison group of term infants without NE, we had 80% power to detect an absolute difference in mean log cytokine levels between NE and non-NE infants ranging from 0.32 to 0.90, at 5% significance level, for the various cytokines.

## Results

**Fig. 1:**
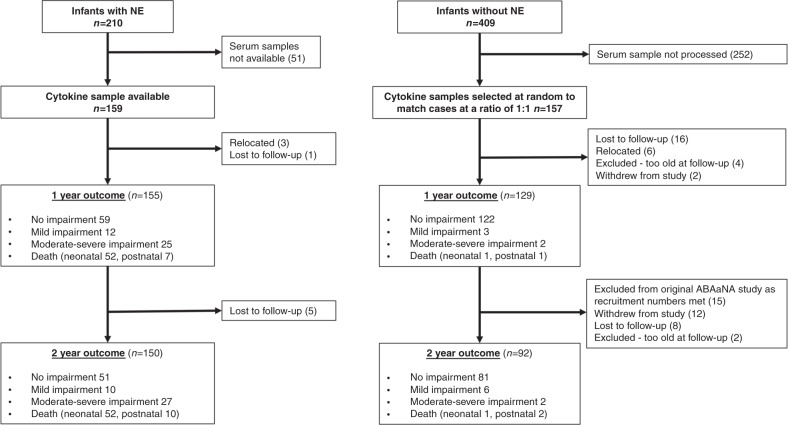
Study Participants. Flow diagram of participants and neurodevelopmental outcomes.

During the 13-month recruitment period (September 2011 to October 2012), 36,926 infants were born at MNRH, from whom 210 with NE and 409 without NE were recruited to the original (parent) ABAaNA study. Serum samples, taken on recruitment, were processed for all NE participants with available stored serum samples (*n* = 159) and a random sample of 157 non-NE participants who act as a control group for this biomarker sub-study. The flow of participants through this nested substudy is summarized in Fig. 1. No differences were seen in baseline characteristic between those from the parent study who did and did not have cytokine data.

In the parent study, neurodevelopmental follow-up at 2 years were undertaken for infants without NE, selected at a ratio of two non-NE infants to one surviving NE child, resulting in 53 control infants deliberately excluded. Sixteen infants with cytokine data fell within this group and were not included in analyses relating cytokines to later outcomes.

### Cytokine profile in NE infants compared to controls

Blood samples for cytokine concentrations were taken at a mean age of 3.4 h (SD 2.44 h) in the NE cohort and 6.7 h (SD 2.19 h) in infants without NE (*p* < 0.001). Crude analysis of the cytokine profile demonstrated significant differences in TNFα, VEGF, and IL-10 levels between the NE and non-NE infants (Table 1). Following linear regression analysis, adjusting for time of cytokine blood sampling and infant sex, VEGF, IL-6, and IL-10 remained significantly different between groups (Table 1). NE was associated with elevated serum IL-6 (*p* = 0.017) and IL-10 (*p* < 0.001) levels compared to non-NE infants (Table 1). In contrast, VEGF was lower in infants with NE compared to non-NE infants (*p* < 0.001). In crude analysis, higher TNF levels were observed in NE infants (*p* = 0.001) compared to non-NE infants; however, significance was lost following adjusting for variables with linear regression.

**Table 1:** Associations between cytokine levels and neonatal encephalopathy.

Cytokine	NE (*n* = 159), median (IQR)	Non-NE (*n* = 157), median (IQR)	*p* value^a^	Adjusted geometric mean ratio (95% CI)^b^	*p* value
TNFα	5.24 (2.49–10.0)	7.76 (4.15–15.0)	**0.001**	0.81 (0.64, 1.04)	0.093
IL-8	260 (46.9–1138)	285 (30.0–1179)	0.910	0.97 (0.48, 1.95)	0.931
VEGF	91.6 (16.6–201)	203 (82.1–367)	**<0.001**	0.33 (0.22, 0.49)	**<0.001**
IL-1α	2.33 (0.05–5.49)	3.22 (0.05–7.90)	0.204	0.79 (0.57, 1.08)	0.142
IL-6	24.6 (8.43–81.1)	18.5 (7.19–52.1)	0.096	1.69 (1.10, 2.58)	0.017
IL-10	6.72 (0.58–24.5)	0.97 (0–3.17)	**<0.001**	2.93 (2.07, 4.15)	**<0.001**

Data shown are median serum cytokine levels in pg/ml (interquartile range).

Bold values indicate statistical significance *p* < 0.05.

*NE* neonatal encephalopathy, *TNFα* tumor necrosis factor alpha, *IL* interleukin, *VEGF* vascular endothelial growth factor.

^a^Mann–Whitney *U* test.

^b^Linear regression analysis with geometric mean difference in cytokine levels (pg/ml) and 95% confidence intervals (95% CIs) between NE and non-NE infants, adjusted for neonatal sex and time of cytokine blood sample.

### Cytokine profile in NE infants with perinatal inflammation/infection risk factors

We compared cytokine levels among NE infants with and without evidence of perinatal infection/inflammation and observed higher levels of IL-1α in NE infants (*p* = 0.013) with exposure to histological funisitis compared to NE infants without exposure (Table 2). No differences in the remaining cytokine levels were observed among NE infants with and without indicators of perinatal inflammation/infection (Table 2).

**Table 2:** Associations between cytokine levels of NE infants and perinatal inflammation and infection.

	Neonatal bacteremia (*N*^a^ = 14/159)		Raised neonatal CRP^b^ (*N*^a^ = 12/158)		Histological funisitis (*N*^a^ = 12/45)		Raised maternal CRP^c^ (*N*^a^ = 17/156)	
	Adjusted geometric mean ratio (95% CI)	*p* value	Adjusted geometric mean ratio (95% CI)	*p* value	Adjusted geometric mean ratio (95% CI)	*p* value	Adjusted geometric mean ratio (95% CI)	*p* value
TNFα	0.98 (0.55, 1.77)	0.951	1.27 (0.68, 2.37)	0.446	1.79 (0.85, 3.81)	0.125	1.10 (0.77, 1.57)	0.597
IL-8	0.96 (0.18, 5.00)	0.959	0.89 (0.15, 5.15)	0.895	1.81 (0.32, 10.28)	0.493	0.54 (0.20, 1.47)	0.223
VEGF	0.58 (0.20, 1.71)	0.325	2.19 (0.70, 6.82)	0.176	1.35 (0.38, 4.78)	0.632	1.23 (0.63, 2.40)	0.540
IL-1α	1.63 (0.80, 3.31)	0.180	1.00 (0.47, 2.14)	0.999	2.88 (1.27, 6.58)	**0.013**	1.19 (0.77, 1.83)	0.435
IL-6	0.58 (0.20, 1.67)	0.308	1.36 (0.44, 4.24)	0.591	2.54 (0.82, 7.87)	0.103	0.68 (0.35, 1.31)	0.249
IL-10	0.67 (0.26, 1.72)	0.399	0.89 (0.33, 2.44)	0.824	0.78 (0.20, 3.07)	0.719	0.99 (0.55, 1.78)	0.961

Multivariable linear regression analysis; differences are shown as the geometric mean ratio in cytokine levels (pg/ml) with 95% confidence intervals (95% CIs), adjusted for infant sex and time of cytokine blood sampling.

Bold values indicate statistical significance *p* < 0.05.

^a^*N* denotes the number of positive results out of the total numbers of test results available. Within this nested cohort of NE infants with cytokine data, blood culture and qPCR data were available for all infants (*n* = 159), neonatal CRP was available for 158 infants, placental histology data were available for 45 NE cases, and maternal CRP data were available in 156 NE cases.

^b^Defined as neonatal CRP > 97th centile among ABAaNA study non-NE infants (CRP > 31.7 mg/l).

^c^Defined as maternal CRP > 90th centile among mothers of ABAaNA study non-NE infants (CRP > 86.6 mg/l) as previously described.^17^

### Neonatal outcomes

#### Severity of encephalopathy

**Fig. 2:**
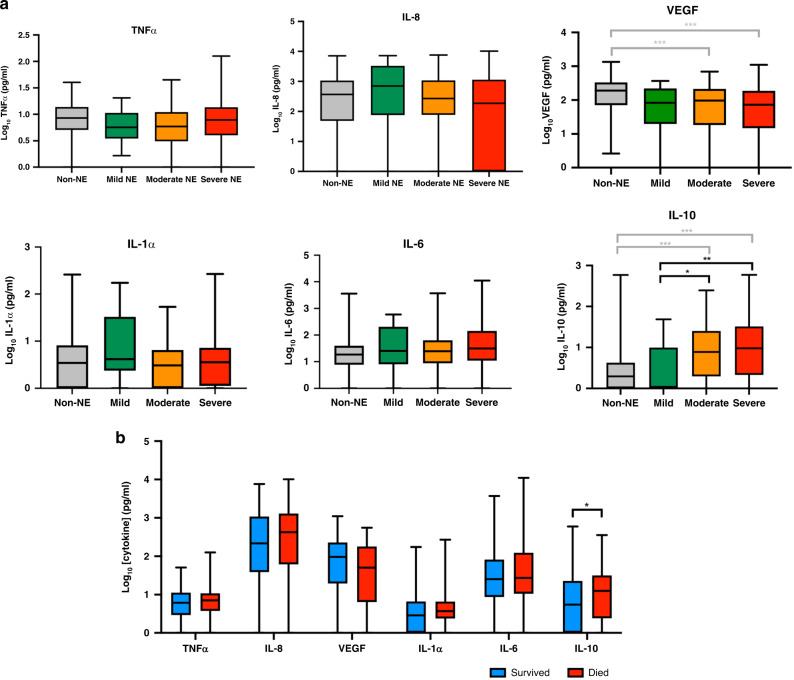
Cytokine profiles and neonatal outcomes. **a** Comparison of cytokine levels (pg/ml) between non-NE infants and NE infants by severity of NE (according to the highest modified Sarnat score in the first 5 days of life). **b** Comparison of cytokine levels (pg/ml) between infants with NE who survived or died in the first 28 days of life. Box plots showing the median, interquartile range, and range of the data. Differences between groups were compared using Kruskal–Wallis test *p < 0.05, **p < 0.01, and ***p < 0.001.

Encephalopathy grades were: 15 mild (9.4%), 87 moderate (54.7%), and 57 severe (35.8%), based on the highest modified Sarnat score over the first 5 days after birth. Median cytokine levels of non-NE infants and by severity of encephalopathy for NE infants are shown in Fig. 2. IL-10 serum levels were higher in both moderate and severely encephalopathic infants compared to the non-NE cohort (*p* < 0.001). We also observed a trend with severity; IL-10 levels were higher in infants with moderate (*p* = 0.046) and severe (*p* = 0.009) encephalopathy compared to infants with mild encephalopathy. We observed lower VEGF serum concentrations in infants with moderate (*p* < 0.001) and severe encephalopathy (*p* < 0.001) compared with non-NE infants. No difference in VEGF levels were observed between the NE severity groups. Serum TNFα, IL-8, IL-1α, and IL-6 did not differ between the groups.

#### Neonatal fatality

Fifty-two (32.7%) infants with NE died in the first 28 postnatal days compared to 1 (0.6%) infant in the non-NE group. Crude serum cytokine levels for infants with NE who survived compared to those who died in the first 28 days are shown in Fig. 2b. After adjustment for time of cytokine blood sampling and infant sex using a multivariable logistic regression model, IL-10 levels doubled the odds of neonatal fatality (adjusted odds ratio (aOR) 2.02, 95% confidence interval (CI) 1.22–3.38, *p* = 0.007). All other cytokine levels were not discriminatory in predicting mortality.

### Early childhood outcomes

Two-year neurodevelopmental outcome data were available for 150 NE infants and 92 non-NE infants. In addition, 1-year (12–15 months) outcome data were available for a further 37 non-NE infants. To ensure consistency between the 1- and 2-year outcome data, a random sample of 37 non-NE infants with both 1- and 2-year outcome data available were assessed. Data between the two time points correlated; all non-NE infants had favorable neurodevelopmental outcomes in both 1- and 2-year data. Therefore, where 2-year data were not available, 1-year outcome data were used and early childhood outcome data in the non-NE group totaled 129 infants.

**Fig. 3:**
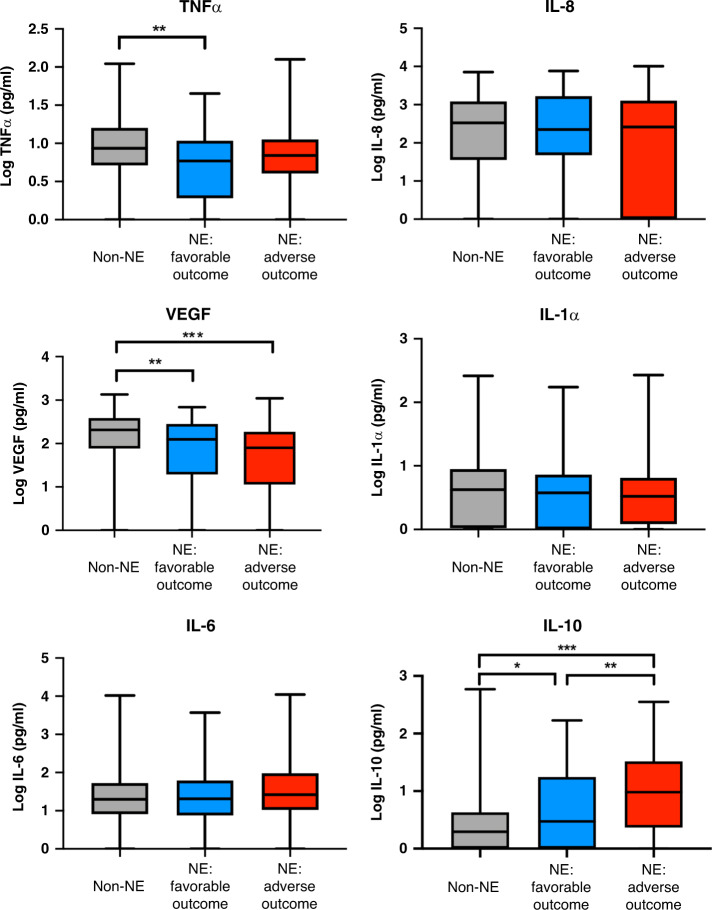
Cytokine profiles and early childhood outcomes. Cytokine levels (pg/ml) in non-NE infants, NE infants with favorable outcomes, and NE infants with adverse outcomes. Adverse outcomes defined as death or neurodevelopmental impairment (global DQ < 70 on GMDS and/or HINE score <67 and/or diagnosis of CP at 1–2 years). Box plots showing the median, interquartile range, and range of the data. Differences between groups were compared using Kruskal–Wallis test where *p < 0.05, **p < 0.01, and ***p < 0.001.

Adverse early childhood outcomes, defined as moderate to severe impairment or death, occurred in 89 of the 150 (59.3%) infants with NE and 4 of the 129 (3.1%) infants without NE. Cytokine profiles for non-NE infants and NE infants with favorable or adverse outcomes are shown in Fig. 3. NE infants with adverse outcomes had higher levels of IL-10 compared to NE infants with favorable outcomes (*p* = 0.005) who in turn had higher levels of IL-10 than infants without NE (*p* = 0.04). On multivariable logistic regression analysis, IL-10 was a predictor of adverse early childhood outcomes (aOR 2.28, 95% CI 1.35–3.86, *p* = 0.002; Table 3). We also observed lower VEGF levels in both NE groups when compared to infants without NE and higher TNFα levels in NE infants with favorable outcome compared to non-NE infants (Fig. 3); however, these were not strongly discriminatory in predicting 2-year adverse outcomes in the adjusted analysis (*p* = 0.05; Table 3).

**Table 3:** Multivariable logistic regression analysis of cytokines to predict adverse early childhood outcomes among infants with NE.

	Unadjusted OR (95% CI)	*p* value	Adjusted OR^a^ (95% CI)	*p* value
TNFα	2.18 (1.01, 4.68)	**0.047**	2.25 (1.00, 5.07)	0.050
IL-8	0.913 (0.71, 1.18)	0.489	0.933 (0.71, 1.22)	0.610
VEGF	0.726 (0.48, 1.10)	0.127	0.748 (0.49, 1.15)	0.185
IL-1α	0.971 (0.53, 1.78)	0.923	1.05 (0.56, 1.96)	0.888
IL-6	1.26 (0.83, 1.92)	0.276	1.17 (0.76, 1.81)	0.475
IL-10	2.11 (1.28, 3.48)	**0.003**	2.28 (1.35, 3.86)	**0.002**

Adverse outcome was defined as death or neurodevelopmental impairment (global DQ < 70 on GMDS and/or HINE score <67 and/or diagnosis of CP at 1–2 years). Results show the OR for adverse outcome per unit increase in log_10_ (cytokine levels).

Bold values indicate statistical significance *p* < 0.05.

^a^Adjusted for sex and time of blood sampling.

**Fig. 4:**
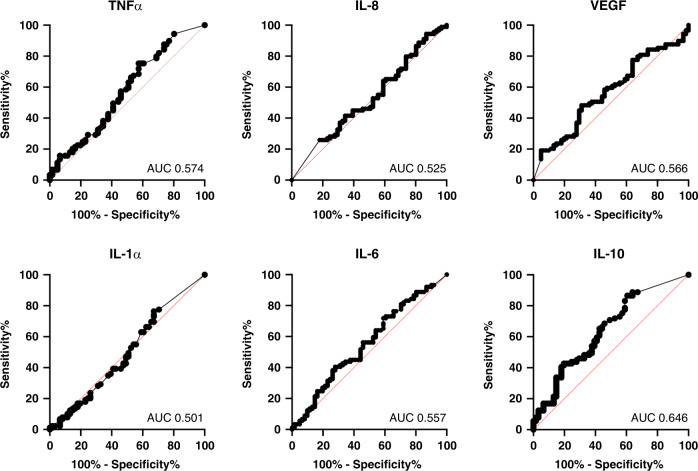
Predictive value of cytokines for early childhood outcomes in NE. ROC curve analysis of cytokine profiles in infants with NE to predict adverse early childhood outcomes.

Outcomes in infants with NE are shown in Fig. 4. IL-10 demonstrated an area under the curve (AUC) value of 0.646 (95% CI 0.555–0.736, *p* = 0.003). Using a cut-off log_10_ IL-10 value of 0.225, for a prevalence of 59.3% of adverse outcome, the sensitivity and specificity were 88.8 and 36.1%, respectively, with a positive predictive value of 66.9% and negative predictive value of 82.4%. All other cytokine values had AUC values close to 0.5 with corresponding *p* > 0.05.

## Discussion

We report selected inflammatory cytokine profiles in the first 12 h after birth in a large cohort of babies with and without NE from a sub-Saharan African hospital setting in Kampala, Uganda. We observed that serum concentrations of IL-10, IL-6, and VEGF were associated with a clinical diagnosis of NE and that serum IL-10 levels within a mean of 3.4 h after birth were higher in moderate–severe NE (based on the worst daily modified Sarnat score) compared to mild NE and no NE. Elevated serum IL-10 levels predicted neonatal mortality (<28 days after birth) and adverse early neurodevelopmental outcomes at 2 years, independent of evidence for perinatal infection/inflammation. Using a cut-off of 0.225 for serum log_10_ (IL-10), the sensitivity and specificity for predicting adverse early childhood outcomes (mortality or NDI) at 2 years was 88.8 and 36.1%, respectively.

NE was associated with elevated IL-6 and IL-10 and reduction in VEGF levels compared to non-NE infants. Cytokines are released by microglia, astrocytes, neurons and systemic immune cells in response to hypoxia–ischemia, modulating the inflammatory response to brain injury.^27^ Past studies of cytokine profiles in NE infants have reported variable, often conflicting, results but have been limited by small sample sizes. A recent small case–control study identified a panel of 17 out of 40 measured cytokines and chemokines, including IL-10, which were elevated within the first 6 h of life in infants with NE compared with healthy term controls.^28^ However, that study did not find an association between NE and IL-6 or VEGF. By contrast a number of observational studies have demonstrated elevated serum IL-6 in infants with NE in the first days after birth compared to non-NE infants.^29–34^ Serum VEGF has previously been demonstrated to be lower in infants with NE compared with controls from serial blood samples taken over the first 24 h after birth.^35^

We have previously reported perinatal infection and inflammation as independent risk factors for NE in this Ugandan population.^17^ Biomarkers to detect NE infants with contributing perinatal infection are sought in order identify infants at greater risk of poor outcome and, in the future, to tailor therapy. In this study, we observed IL-1α levels were higher in NE infants exposed to histological funisitis compared to NE infants without such exposure. IL-1α is a potent pro-inflammatory cytokine. Levels of IL-1 are elevated in amniotic fluid of women with chorioamnionitis^36^ and have been directly implicated in the fetal inflammatory response.^37,38^ Despite the association with funisitis, serum IL-1α was not predictive of short- or long-term outcome after NE in our cohort. It is possible that the association between IL-1α and funisitis is a chance finding due to multiplicity. No other measured serum cytokines were able to discriminate infection or inflammation exposure among infants with NE.

We observed that IL-10 levels was elevated in moderate and severe NE, compared to mild NE and non-NE infants, and associated with adverse early childhood outcomes. The associations between raised IL-10 and NE severity,^39^ neonatal mortality,^40,41^ and early childhood NDI in NE^42^ have been reported previously in several smaller studies. IL-10 is an anti-inflammatory cytokine released via a Toll-like receptor 2/nuclear factor-κB-dependent pathway^43,44^ following HI.^45^ Whether elevated IL-10 reflects, or contributes to, severity of brain injury is unclear. Traditionally, pro-inflammatory responses have been implicated in neuronal injury and anti-inflammatory cytokines associated with inflammation resolution, neuronal survival, and repair.^5^ Paradoxically, there is preclinical evidence that IL-10 may exacerbate brain injury.^46^ Clinically, IL-10 has also been implicated in *immunoparalysis*, a term to describe the pathologically blunted inflammatory response resulting from a persistence of marked compensatory anti-inflammatory signaling.^47^ An association between immunoparalysis and adverse outcomes has been observed in the pediatric intensive care setting following cardiopulmonary bypass,^46^ sepsis, and trauma.^48^ In cooled infants with NE, chemokine-mediated leukopenic immunoparalysis was observed in infants with poor outcome at 12 months (death or NDI).^49^ In vitro studies report the inhibition of pro-inflammatory TNFα production by IL-10.^50,51^ In our study, we observed that TNFα was not elevated among infants with NE, even in association with perinatal infection and inflammation. The balance between a pro-inflammatory and anti-inflammatory phenotype is complex and likely key to neurogenesis and repair following neonatal brain injury during the period of tertiary brain injury.^52,53^ Importantly, altered inflammatory response in the neonatal period may have longer-term implications. Recently, Zareen et al. showed altered cytokine response persisting into school age after NE; IL-10 levels remained elevated in infants with NE and hyporesponsiveness to lipopolysaccharide stimulation was observed in several cytokines, including IL-10.^54^ Neuroprotective interventions targeted at modulating the inflammatory response may further improve outcomes in infants with NE. In preclinical studies, mesenchymal stem cell therapy administered to rats subjected to HI injury was associated with a reduction in IL-10 levels, reduction in brain cell death, and improved learning memory function.^55^

Adverse neurodevelopmental outcome after NE was common in this population, affecting nearly one-third of NE survivors.^18^ Neuroprotective interventions available for infants with NE in low-income settings are limited. The benefits of therapeutic hypothermia remains uncertain in this setting.^56,57^ Animal studies suggest that cooling may not be neuroprotective in combination with infection and inflammation,^58^ whereas clinical studies showed more variable findings.^59,60^ A large multi-center randomized controlled trial in LMICs is currently underway, but these results may not be generalizable to sub-Saharan Africa.^61^ Identifying biomarkers to risk stratify for early intervention and future neuroprotective trials are urgently needed. Here we demonstrated that IL-10 can predict adverse neurodevelopmental outcome with a reasonable sensitivity of 88.8% and negative predictive value of 82.4% using a log_10_ IL-10 threshold value of 0.225. We opted for a cut-off value that maximizes the identification of at-risk infants and reduces false negative results as it is important in this setting that we avoid missing infants who may benefit the most from early interventional programs^62^ and future neuroprotective therapies. Inflammatory cytokines have been explored as potential biomarkers for NE; however, their predictive accuracy has varied across different studies.^12^ The temporal evolution of cytokines after HI injury^35,42,63^ may in part explain this variability. In a study of cytokines nested within a randomized control trial investigating erythropoietin as an adjunct agent with therapeutic hypothermia for NE, IL-10 at 16.2 h showed a modest correlation with global brain injury score on MRI; however, no correlation was observed when IL-10 was measured on day 5.^13^ In the piglet model of HI, Rocha-Ferreira et al.^64^ showed that serum IL-10 levels peaked at 36 h after HI followed by a gradual decline. IL-10 correlated with magnetic resonance spectroscopy-measured lactate/*N*-acetyl aspartate peak ratio, a robust biomarker of outcomes in infants with NE.^11^ A biphasic response in IL-6 exists as seen in both clinical^42^ and preclinical studies,^64^ with a secondary rise at 36–48 h, which may explain the poor correlation in IL-6 with outcomes observed in this study. Similarly, VEGF levels evolves in the first four days of life and its correlation with NE severity varies according to time of cytokine measurement.^65^ Serial measurement of cytokine may further improve accuracy to predict outcomes and therefore requires further investigation.

The study has several limitations. Cytokine data were only available for a single time point. Serial cytokine data correlated with outcomes may provide further insight into the evolution of the neuroinflammatory profile of babies with NE and the optimal timing of measurement to provide the best predictive value as a biomarker. Placental histology was examined in only a quarter of cases, leading to potential under-representation of chorioamnionitis in this cohort, which may be a confounding factor in cytokine expression. Histological chorioamnionitis was used rather than clinical chorioamnionitis as histology more reliably predict intrauterine inflammation.^66,67^ Although infants who received antibiotics prior to enrollment were excluded from the parent study, this was uncommon and unlikely to have introduced bias. While the diagnosis of neonatal bacteremia was strengthened by using species-specific bacterial PCR in addition to blood cultures, under-diagnosis cannot be excluded. Although assessment of a random sample of infants showed a good correlation between 1- and 2-year outcomes, later childhood outcomes at school age would enable exploration of associations with longer-term outcomes.

## Conclusion

Inflammatory cytokines may induce, modify, and indicate NE severity and outcome. In this cytokine substudy nested within the ABAaNA study,^17^ a prospective unmatched case–control study investigating perinatal risk factors for NE in Uganda, serum IL-10 levels at 3 h were significantly elevated in infants with NE and predicted neonatal mortality and adverse neurodevelopmental outcomes at 2 years. IL-10 is an anti-inflammatory cytokine and may have a role in modulating brain injury long term through the nature of the pro-inflammatory response to HI. Serum IL-10 shows promise as a biomarker to predict adverse early childhood outcomes and may be useful in future neuroprotection trials targeted to infants at the highest risk of death or NDI.

## Supplementary information

Ethical Approval

STROBE checklist v4 combined[2]
