# Supplementary material for: Elevated serum IL-10 is associated with severity of neonatal encephalopathy and adverse early childhood outcomes
Source: Pediatr Res. 2021 Mar 5;92(1):180–9. doi: 10.1038/s41390-021-01438-1 (PMC9411052; doi:10.1038/s41390-021-01438-1)
Supplement: Supplementary file 1 — Ethical Approval [file 41390_2021_1438_MOESM1_ESM.pdf]

UCL RESEARCH ETHICS COMMITTEE
GRADUATE SCHOOL OFFICE

Professor Nicola Robertson
Institute for Women's Health
2nd Floor, Medical School Building
Room 239, 74 Huntley Street
UCL

18 July 2012

Dear Professor Robertson

Notification of Ethical Approval
Project ID: 4160/001: Early neurodevelopmental outcomes and cerebral palsy after neonatal encephalopathy in Uganda: an assessment of the role of perinatal infection/inflammation

I am pleased to confirm that your study has been approved by the UCL Research Ethics Committee for the duration of the project i.e. until May 2014.

Approval is subject to the following conditions:

1. You must seek Chair's approval for proposed amendments to the research for which this approval has been given. Ethical approval is specific to this project and must not be treated as applicable to research of a similar nature. Each research project is reviewed separately and if there are significant changes to the research protocol you should seek confirmation of continued ethical approval by completing the 'Amendment Approval Request Form'.

The form identified above can be accessed by logging on to the ethics website homepage: http://www.grad.ucl.ac.uk/ethics/ and clicking on the button marked 'Key Responsibilities of the Researcher Following Approval'.

2. It is your responsibility to report to the Committee any unanticipated problems or adverse events involving risks to participants or others. Both non-serious and serious adverse events must be reported.

   Reporting Non-Serious Adverse Events
   For non-serious adverse events you will need to inform Helen Dougal, Ethics Committee Administrator (ethics@ucl.ac.uk), within ten days of an adverse incident occurring and provide a full written report that should include any amendments to the participant information sheet and study protocol. The Chair or Vice-Chair of the Ethics Committee will confirm that the incident is non-serious and report to the Committee at the next meeting. The final view of the Committee will be communicated to you.

   Reporting Serious Adverse Events
   The Ethics Committee should be notified of all serious adverse events via the Ethics Committee Administrator immediately the incident occurs. Where the adverse incident is unexpected and serious, the Chair or Vice-Chair will decide whether the study should be terminated pending the opinion of an independent expert. The adverse event will be considered at the next Committee meeting and a decision will be made on the need to change the information leaflet and/or study protocol.

On completion of the research you must submit a brief report (a maximum of two sides of A4) of your findings/concluding comments to the Committee, which includes in particular issues relating to the ethical implications of the research.

With best wishes for the research.

Yours sincerely

Professor John Foreman
Chair of the UCL Research Ethics Committee

Cc: Dr Cally Tann

UCL Research Ethics Committee, c/o The Graduate School, North Cloisters, Wilkins Building
University College London Gower Street London WC1E 6BT
Tel: +44 (0)20 7679 7844 Fax: +44 (0)20 7679 7043
ethics@ucl.ac.uk
www.ucl.ac.uk/gradschool

LONDON SCHOOL OF HYGIENE
& TROPICAL MEDICINE

ETHICS COMMITTEE

APPROVAL FORM
Application number: 5892

Name of Principal Investigator	**Dr Cally Tann**
Faculty	**Infectious and Tropical Diseases**
Head of Faculty	**Professor Simon Croft**

Title: **Associations between Birth Asphyxia and infection amongst Newborns in Africa: Perinatal risk factors for neonatal encephalopathy in Uganda (the ABAaNA study)**

This application is approved by the Committee.

Chair of the Ethics Committee ......

Date ....07 February 2011……………………………….

Approval is dependent on local ethical approval having been received.

Any subsequent changes to the application must be submitted to the Committee via an E2 amendment form.

London School of Hygiene & Tropical Medicine
Keppel Street, London WC1E 7HT
United Kingdom
Switchboard: +44 (0)20 7636 8636

www.lshtm.ac.uk

LONDON SCHOOL of HYGIENE &TROPICAL MEDICINE

Observational / Interventions Research Ethics Committee

Cally Tann
Honorary Clinical Lecturer
CR / ITD
LSHTM

10 February 2014

Dear Dr. Tann,

Study Title: **Associations between Birth Asphyxia and infection amongst Newborns in Africa: Perinatal risk factors for neonatal encephalopathy in Uganda (the ABAaNA study)**
LSHTM ethics ref: **5892**
LSHTM amend no: **A488**

Thank you for your letter of 31 January 2014, responding to the Observational Committee's request for further information on the above amendment to research and submitting revised documentation.

The further information has been considered on behalf of the Committee by the Chair.

Confirmation of ethical opinion

On behalf of the Committee, I am pleased to confirm a favourable ethical opinion for the above amendment to research on the basis described in the application form, protocol and supporting documentation as revised, subject to the conditions specified below.

Conditions of the favourable opinion

Approval is dependent on local ethical approval for the amendment having been received, where relevant.

Approved documents

The final list of documents reviewed and approved by the Committee is as follows:

Document	Version	Date
LSHTM amendment application	n/a	
ABAaNA study protocol	9	Dec 2013
ABAaNA study protocol appendix 1	2	Nov 2013
ABAaNA study protocol appendix 2	1	Dec 2013
Information consent form: cases	1	Dec 2013
Information consent form: controls	1	Dec 2013
Consent for photographs	1	Dec 2013

After ethical review

Any further changes to the application must be submitted to the Committee via an Amendment form on the ethics online applications website. The Principal Investigator is reminded that all studies are also required to notify the ethics committee of any serious adverse events which occur during the project via an Adverse Event form on the ethics online applications website. At the end of the study, please notify the committee via an End of Study form on the ethics online applications website. Link to the ethics online applications website: http://leo.lshtm.ac.uk

Yours sincerely,

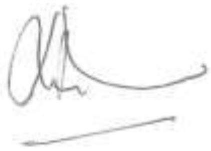

Professor John DH Porter
Chair
ethics@lshtm.ac.uk
http://www.lshtm.ac.uk/ethics/

Uganda National Council for Science and Technology

(Established by Act of Parliament of the Republic of Uganda)

UNCST

Our Ref: HS 945 **June 2, 2011**

Dr. Cally Tann
C/O MRC/UVRI Research Unit on AIDS
Uganda Virus Research Institute
P.O Box 49
Entebbe

Dear Dr. Tann,

RE: RESEARCH PROJECT, "ASSOCIATION BETWEEN BIRTH ASPHYXIA AND INFECTION AMONGST NEWBORNS IN AFRICA: PERINATAL RISK FACTORS FOR NEONATAL ENCEPHALOPATHY IN UGANDA (ABAaNA STUDY)"

This is to inform you that the Uganda National Council for Science and Technology (UNCST) approved the above research proposal on **May 10, 2011.** The approval will expire on **May 10, 2012.** If it is necessary to continue with the research beyond the expiry date, a request for continuation should be made in writing to the Executive Secretary, UNCST.

Any problems of a serious nature related to the execution of your research project should be brought to the attention of the UNCST, and any changes to the research protocol should not be implemented without UNCST's approval except when necessary to eliminate apparent immediate hazards to the research participant(s).

This letter also serves as proof of UNCST approval and as a reminder for you to submit to UNCST timely progress reports and a final report on completion of the research project.

Yours sincerely,

Leah Nawegulo
for: Executive Secretary
UGANDA NATIONAL COUNCIL FOR SCIENCE AND TECHNOLOGY

LOCATION/CORRESPONDENCE

Plot 6 Kimera Road, Ntinda
P. O. Box 6884
KAMPALA, UGANDA

COMMUNICATION

TEL: (256) 414 705500
FAX: (256) 414-234579
EMAIL: info@uncst.go.ug
WEBSITE: http://www.uncst.go.ug

Uganda National Council for Science and Technology

(Established by Act of Parliament of the Republic of Uganda)

UNCST

Our Ref: HS 945 **2nd October 2012**

Dr. Cally Tann
MRC/UVRI Research Unit on AIDS
Uganda Virus Research Institute
Entebbe

Dear Dr. Tann,

RE: RESEARCH PROJECT, "ASSOCIATION BETWEEN BIRTH ASPHYXIA AND INFECTION AMONGST NEWBORNS IN AFRICA: PERINATAL RISK FACTORS FOR NEONATAL ENCEPHALOPATHY IN UGANDA"

This is to inform you that on 13th September 2012, Uganda National Council for Science and Technology (UNCST) reviewed and approved the revisions made to the above study.

The approved changes include:

1. Extension of the follow-up of the ABAaNA cohorts to 12-15 months of age.
2. Addition of Herpes Simplex Virus to the Spectrum of PCR's performed on neonatal blood.

The approval covers the following attachments:

a. The ABAaNA Study protocol (version 6) dated July 2012.
b. Appendix 1: Neurodevelopment outcomes at one year (version 1) dated July 2012.
c. ABAana Study: Early Neurodevelopment outcomes (Cases) Information Consent dated July 2012.
d. ABAana Study: Early Neurodevelopment outcomes (Controls) Information Consent dated July 2012.
e. ABAana Study: Maternal Experiences at one year Information Consent dated July 2012.
f. Griffiths Mental Development Scales-Extended Revised Record Book.

The study is valid until 10th May 2013. If however, it is necessary to continue with the study beyond the expiry date, a request for continuation should be made to the Executive Secretary, UNCST

Yours sincerely,

Leah Nawegulo
for: Executive Secretary
UGANDA NATIONAL COUNCIL FOR SCIENCE AND TECHNOLOGY

LOCATION/CORRESPONDENCE

Plot 6 Kimera Road, Ntinda
P. O. Box 6884
KAMPALA, UGANDA

COMMUNICATION

TEL: (256) 414 705500
FAX: (256) 414-234579
EMAIL: info@uncst.go.ug
WEBSITE: http://www.uncst.go.ug

Uganda National Council for Science and Technology

(Established by Act of Parliament of the Republic of Uganda)

UNCST

Our Ref: HS 945 **4th March 2014**

Dr. Cally Tann
MRC/UVRI Research Unit on AIDS
Uganda Virus Research Institute
Entebbe

Dear Dr. Tann,

RE: RESEARCH PROJECT, "ASSOCIATION BETWEEN BIRTH ASPHYXIA AND INFECTION AMONGST NEWBORNS IN AFRICA: PERINATAL RISK FACTORS FOR NEONATAL ENCEPHALOPATHY IN UGANDA"

This is to inform you that on 3rd March 2014, Uganda National Council for Science and Technology (UNCST) reviewed and approved the revisions made to the above study.

The approved changes include:

1. Seeing children at again 27-30 months.
2. Examining stored blood for some additional neonatal cytokines that are signs of newborn response to infection.

The approval covers the following attachments:

a. The ABAaNA Study protocol (version 9 dated Dec 2013).
b. Neurodevelopment outcomes at Two years (Cases) Information: Consent version 1dated Dec 2013.
c. Neurodevelopment outcomes at Two years (Controls) Information: Consent version 1 dated Dec 2013.
d. Consent to photograph/Medical Imaging: ABAaNA at TWO, dated Jan 2014.
e. Appendix 1: Neurodevelopment outcomes at one year, version 2 dated Nov 2013.
f. Appendix 2: Neurodevelopment outcomes at two years, version 2 dated Jan 2014

The study is valid until 10th May 2014. If however, it is necessary to continue with the study beyond the expiry date, a request for continuation should be made to the Executive Secretary, UNCST

Yours sincerely,

Leah Nawegulo Omongo
for: Executive Secretary
UGANDA NATIONAL COUNCIL FOR SCIENCE AND TECHNOLOGY

LOCATION/CORRESPONDENCE

Plot 6 Kimera Road, Ntinda
P. O. Box 6884
KAMPALA, UGANDA

COMMUNICATION

TEL: (256) 414 705500
FAX: (256) 414-234579
EMAIL: info@uncst.go.ug
WEBSITE: http://www.uncst.go.ug

Tel: (256) 41- 321962 (Direct)
(256) 41- 320385/6 (General)
Fax: (256) 41- 320483
Email:directoruvri@ug.cdc.gov

FOR GOD AND MY COUNTRY
THE REPUBLIC OF UGANDA

UGANDA VIRUS RESEARCH INSTITUTE
P.O.Box 49 ENTEBBE (U)

Our Ref : GC/127/11/03/10
Your Ref:

1st March 2011

Dr. Cally Tann,

RE: UVRI SEC review of protocol titled: **"Associations between Birth Asphyxia and infection amongst Newborns in Africa".**

Thank you for your response to the queries addressed to you during the SEC meeting of January 20th 2011.

This is to inform you that your responses were reviewed during the SEC meeting of 24th February 2011 and met the requirements of the UVRI Science and Ethics Committee.

UVRI SEC annual approval has been given for you to conduct your research up to 28th February 2012. Annual progress report and request for extension should be submitted to UVRI SEC prior to the expiry date, to allow timely review.

You can now commence with your study after registration with the Uganda National Council for Science and Technology (UNCST).

Note: UVRI SEC requires you to submit a copy of the UNCST approval letter for the above study before commencement.

Yours sincerely,

Tom Lutalo
Chair, UVRI SEC

C.C Secretary, UVRI SEC

UNHRO
Uganda National Health
Research Organisation

Uganda Virus Research Institute

Plot 51-59, Nakiwogo Road, Entebbe
P.O. Box 49, Entebbe-Uganda
Tel: +256 414 320 385 / 6
Fax: +256 414 320 483
Email: directoruvri@uvri.go.ug

REPUBLIC OF UGANDA

Our Ref: GC/127/14/01/219

Your Ref: ……

10th January 2014

Dr. Cally Tann,

RE: UVRI SEC review of protocol amendment titled **"Association between Birth Asphyxia and infection amongst Newborns in Africa: Perinatal risk factors for neonatal encephalopathy in Uganda"**

Thank you for submitting the above study amendment dated 30th December 2013 to the UVRI Science and Ethics Committee (SEC).

This is to inform you that the amendment to the above named protocol was reviewed and met the requirements of the UVRI Science and Ethics Committee. UVRI SEC approval has been given for you to continue with the proposed amendment.

The reviewed and approved amendment is;

- Seeing children again at two years for more detailed assessment of important early cognitive, language and behavioural outcomes when compared to one year, to provide comparison data to cohorts from other settings and to ensure long term retention of the cohorts.

You can continue with your study after registration of your amendment with the Uganda National Council for Science and Technology (UNCST).

Yours sincerely,

Mr. Tom Lutalo
Chair, UVRI SEC
C.C Secretary, UVRI SEC

UNHRO
Uganda National Health Research Organisation

Uganda Virus Research Institute

Plot 51-59, Nakiwogo Road, Entebbe
P.O. Box 49, Entebbe-Uganda
Tel: +256 414 320 385 / 6
Fax: +256 414 320 483
Email: directoruvri@uvri.go.ug

REPUBLIC OF UGANDA

Our Ref: GC/127/13/11/05

Your Ref: ……

06th November 2013

Dr. Cally Tann,

RE: UVRI SEC review of protocol amendment titled **"Associations between Birth Asphyxia and infection amongst newborns in Africa: Perinatal risk factors for neonatal encephalopathy in Uganda (The ABAaNA study)."**

Thank you for submitting the above study amendment dated 28th October 2013 to the UVRI Science and Ethics Committee (SEC).

This is to inform you that the amendments to the above named protocol were reviewed and met the requirements of the UVRI Science and Ethics Committee. UVRI SEC approval has been given for you to continue with the proposed amendments.

The reviewed and approved amendments include;

1. Examining the stored newborn blood for some additional neonatal cytokines that are signs of the newborn response to infection to indicate which babies are most at risk of newborn brain injury and identify future strategies for intervention.
2. Addition made to the protocol for the one year follow-up protocol appendix.

You can continue with your study after registration of your amendment with the Uganda National Council for Science and Technology (UNCST).

Yours sincerely,

Mr. Tom Lutalo
Chair, UVRI SEC
C.C Secretary, UVRI SEC

UNHRO
Uganda National Health Research Organisation

Uganda Virus Research Institute
Plot 51-59, Nakiwogo Road, Entebbe
P.O. Box 49, Entebbe-Uganda
Tel: +256 414 320 385 / 6
Fax: +256 414 320 483
Email: directoruvri@uvri.go.ug

REPUBLIC OF UGANDA

Our Ref: GC/127/12/08/08

Your Ref: ......

23rd August 2012

Dr. Cally Tann,

RE: UVRI SEC review of protocol amendment titled **"Associations between Birth Asphyxia and infection amongst Newborns in Africa: Perinatal risk factors for neonatal encephalopathy in Uganda."**

Thank you for submitting the above study amendment dated 31st July 2012 to the UVRI Science and Ethics Committee (SEC).

This is to inform you that your protocol amendment was reviewed and met the requirements if the UVRI Science and Ethics Committee. UVRI SEC approval has been given for you to continue with the proposed amendment.

The reviewed and approved amendments include;

1. Extension of the follow-up of the ABAaNA cohorts to 12-15 months of age.
2. Addition of Herpes Simplex Virus to the spectrum of PCRs being performed on neonatal blood.

You can continue with your study after registration of your amendment with the Uganda National Council for Science and Technology (UNCST).

Mr. Tom Lutalo
Chair, UVRI SEC

C.C Secretary, UVRI SEC

UNHRO
Uganda National Health
Research Organisation

Uganda Virus Research Institute

Plot 51-59, Nakiwogo Road, Entebbe
P.O. Box 49, Entebbe-Uganda
Tel: +256 414 320 385 / 6
Fax: +256 414 320 483
Email: directoruvri@uvri.go.ug

REPUBLIC OF UGANDA

Our Ref: GC/127/14/01/219

Your Ref: ……

10th January 2014

Dr. Cally Tann,

RE: UVRI SEC review of protocol amendment titled **"Association between Birth Asphyxia and infection amongst Newborns in Africa: Perinatal risk factors for neonatal encephalopathy in Uganda"**

Thank you for submitting the above study amendment dated 30th December 2013 to the UVRI Science and Ethics Committee (SEC).

This is to inform you that the amendment to the above named protocol was reviewed and met the requirements of the UVRI Science and Ethics Committee. UVRI SEC approval has been given for you to continue with the proposed amendment.

The reviewed and approved amendment is;

- Seeing children again at two years for more detailed assessment of important early cognitive, language and behavioural outcomes when compared to one year, to provide comparison data to cohorts from other settings and to ensure long term retention of the cohorts.

You can continue with your study after registration of your amendment with the Uganda National Council for Science and Technology (UNCST).

Yours sincerely,

Mr. Tom Lutalo
Chair, UVRI SEC
C.C Secretary, UVRI SEC
